# Risk factors for the development of pneumonia post cardiac surgery

**DOI:** 10.5830/CVJA-2012-005

**Published:** 2012-05

**Authors:** AE Topal, MN Eren

**Affiliations:** Cardiovascular Surgery Department, Dicle University Medical Faculty, Diyarbakır,Turkey; Cardiovascular Surgery Department, Dicle University Medical Faculty, Diyarbakır,Turkey

**Keywords:** cardiac surgery, pneumonia, atrial fibrillation, transfusion, chronic obstructive pulmonary disease

## Abstract

**Objectives:**

Postoperative pneumonia is a devastating complication after cardiac surgery that increases morbidity and mortality. The objective of this study was to identify potential risk factors for the development of nosocomial pneumonia post cardiac surgery by the way of logistic regression analysis.

**Design:**

Data of the last 162 patients undergoing cardiac surgery before November 2009 were retrospectively collected and analysed.

**Results:**

The mean age of the patients was 65.57 ± 10.48 years and 83 (51%) were male. Postoperative pneumonia was diagnosed in 21 (13%) patients. The mean remaining time in the intensive care unit and mean length of hospitalisation were longer for patients with postoperative pneumonia. Pre-operative heart rate, previous diabetes mellitus, previous chronic obstructive pulmonary disease, postoperative urea, creatinine and potassium levels, extubation time, postoperative atrial fibrillation, and number of units of transfused packed red blood cells (pRBC) and fresh frozen plasma were associated with higher occurrence of postoperative pneumonia on univariate analysis.

**Conclusions:**

On logistic regression analysis, pRBC transfusion, previous chronic obstructive pulmonary disease and postoperative atrial fibrillation remained as independent predictors for the development of postoperative pneumonia.

## Abstract

Despite the progress made in surgery and anaesthesia, the risk of developing nosocomial infections remains a real threat as more patients of greater age and with more co-morbidities are operated on.^1^ Particularly cardiac surgery creates a high risk for the development of hospital infections and among these, pneumonia plays an important role as it increases morbidity and mortality by causing pulmonary dysfunction or multi-organ failure.

Patients undergoing cardiovascular operations are currently older and with serious co-morbid disease. Compared to their younger counterparts, heart surgery in elderly patients has been implicated in the higher risk of mortality and recurrent pulmonary complications.^2^ Moreover, emergence of antibiotic-resistant pathogens increases the incidence of refractory pneumonia.

The aim of our study was to identify potential risk factors for the development of nosocomial pneumonia post cardiac surgery and thus contribute to decreasing the incidence of pneumonia by identifying preventable risk factors.

## Methods

This retrospective study was performed on the last 162 patients who underwent cardiac surgery (coronary artery bypass graft surgery, valve-replacement surgery) at our reference centre up to November 2009. The exclusion criteria were usage of immunosuppressive agents and an identifiable infection prior to surgery.

All patients received standardised anaesthetic management. In the operating room, leads II and V5 on the electrocardiogram (ECG) and arterial blood pressure were continuously monitored. Anaesthesia was induced with intravenous midazolam (0.03–0.07 mg/kg), sufentanil (1.5–3.0 mg/kg) and rocuronium bromide (0.9 mg/kg), and maintained with sevoflurane (0.8–1.5%) and continuous infusion of sufentanil (0.5–1.5 mg/kg/h).

All surgical procedures were performed through a median sternotomy. All patients included in the study received prophylactic administration of intravenous cefazolin peri-operatively (1 g intravenously 30 minutes prior to the first incision, every eight hours during surgery and postoperatively for three days).

Pneumonia was considered clinically present as a new radiographic pulmonary infiltrate, consolidate, cavitation or pneumatocele in the presence of the following conditions: fever (> 38°C) without other recognised causes, leucocytosis (> 12 000/μl) or leucopenia (< 4 000/μl) and new-onset purulent sputum with a Gram-positive stain finding.

Possible risk factors and outcomes associated with pneumonia post cardiac surgery were analysed, including pre-operative variables [age, gender, heart rate, mean blood pressure, body surface area, urea, creatinine and potassium levels, co-morbidities, NYHA class, and left ventricular ejection fraction (LVEF)], operative variables [on/off pump surgery, cross-clamp time, cardiopulmonary bypass (CPB) time, total operation time, and need for intra-operative inotropic support], and postoperative variables [extubation time, chest tube drainage, number of units of transfused packed red blood cells (pRBC) and fresh frozen plasma (FFP), urea, creatinine and potassium levels, and postoperative atrial fibrillation (AF)].

## Statistical analysis

The normality of the variables was analysed by Kolmogorov–Smirnov test. Continuous variables are presented as means with standard deviations and were compared among groups using the Student’s *t*-test or Mann-Whitney U-test when appropriate (non-parametric data). Dichotomous variables are presented as percentages and were compared among groups using a Chi-square or Fisher exact test when appropriate.

All variables showing an association (*p* ≤ 0.05) with pneumonia post cardiac surgery were then entered into a forward stepwise multivariate logistic regression model. A two-sided *p*-value < 0.05 was considered significant in the multivariate logistic regression model. Adjusted odds ratios (AORs), 95% confidence intervals (CIs), and two-tailed *p*-values were calculated for all variables retained in the multivariate logistic regression model. Statistical analyses were carried out using the statistical packages for SPSS 15.0 for Windows (SPSS Inc., Chicago, IL, USA).

## Results

The study group comprised 162 patients who underwent cardiac surgery. The mean age of the patients was 65.57 ± 10.48 years (range 43–84 years), and 83 (51%) were male. Of 162 operations, 140 were coronary artery bypass graft (CABG) surgery, and 22 patients underwent valve replacement surgery. Sixteen CABG operations were performed without cardiopulmonary bypass (CPB).

Before surgery, 20 patients were in New York Heart Association (NYHA) functional class I, 101 patients were in class II, and 41 were in class III. Pre-operative co-morbid diseases were diabetes in 106 patients, hypertension in 111 patients, chronic obstructive pulmonary disease (COPD) in 43 patients, peripheral artery disease in 10 patients and AF in 29 patients. Ninety-one (56.2%) patients were tobacco users. The patients’ characteristics and peri-operative variables are shown in Table 1.

**Table 1:** Effect Of Patients’ Characteristics And Peri-Operative Variables On Development Of Pneumonia Post Cardiac Surgery

	*Patients without pneumonia (n = 141)*	*Patients with pneumonia (n = 21)*	p-*value*
Male, *n* (%)	72 (51.1)	11 (52.4)	0.911**
Age (years)	65.3 ± 10.4	67.5 ± 11.0	0.362*
Pre-operative variables			
NYHA class, *n* (%)			0.889**
I	17 (12.1)	3 (14.3)	
II	89 (63.1)	12 (57.1)	
III	35 (24.8)	6 (28.6)	
Ejection fraction (%)	50.5 ± 8.7	47.6 ± 9.4	0.170*
Heart rate (/min)	92.2 ± 6.3	89.2 ± 7.4	0.047*
Mean blood pressure (mmHg)	91.7 ± 9.7	88.3 ± 6.9	0.130*
Body surface area (m^2^)	1.7 ± 0.1	1.7 ± 0.2	0.242*
Urea (mg/dl)	40.2 ± 14.3	44.7 ± 13.1	0.174*
Creatinin (mg/dl)	1.0 ± 0.2	1.0 ± 0.2	0.403*
Potassium (mmol/l)	4.3 ± 0.4	4.2 ± 0.5	0.855*
Hypertension, *n* (%)	97 (68.8)	14 (66.7)	0.845**
Hyperlipidaemia, *n* (%)	89 (63.1)	14 (66.7)	0.753**
Tobacco usage, *n* (%)	79 (56)	12 (57.1)	0.924**
Peripheral arterial disease, *n* (%)	9 (6.4)	1 (4.8)	0.774**
Atrial fibrillation, *n* (%)	25 (17.7)	4 (19)	0.884**
COPD, *n* (%)	27 (19.1)	16 (76.2)	< 0.001**
Diabetes mellitus, *n* (%)	88 (62.4)	18 (85.7)	0.037**
Intra-operative variables			
Off pumpn, *n* (%)	13 (9.2)	3 (14.3)	0.469**
Cross-clamp time (min)	36.2 ± 10.8	37.8 ± 9.8	0.552*
CPB time (min)	60.4 ± 16.9	61.2 ± 15.0	0.859*
Total operation time (min)	114.9 ± 20.3	114.8 ± 15.0	0.971*
Need for inotropic support, *n* (%)	30 (21.3)	8 (38.1)	0.091**
Postoperative variables			
Extubation time (hour)	7.5 ± 2.8	25.0 ± 21.3	< 0.001*
Chest tube drainage (ml)	610.6 ± 286.0	733.3 ± 287.4	0.069*
Units of transfused FFP	2.9 ± 1.5	4.8 ± 3.3	< 0.001*
Units of transfused pRBC	5.8 ± 1.6	10.8 ± 3.3	< 0.001*
Urea (mg/dl)	45.2 ± 15.7	57.8 ± 21.6	0.001*
Creatinine (mg/dl)	1.1 ± 0.4	1.3 ±.5	0.009*
Potassium (mmol/l)	3.7 ± 0.7	4.1 ± 1.0	0.008*
Atrial fibrillation, *n* (%)	25 (17.7)	13 (61.9)	< 0.001**

*Student’s *t*-test, **Fisher’s exact test. COPD: chronic pulmonary obstructive disease, CPB: cardiopulmonary bypass, FFP: fresh frozen plasma, pRBC: packed red blood cells.

Postoperative pneumonia was detected in 21 (13%) patients. Mean remaining time in the intensive care unit and mean length of hospitalisation were longer for patients with postoperative pneumonia compared to the patients without postoperative pneumonia (4.5 ± 2.7 vs 3.1 ± 1.1 days, *p* < 0.001; 13.1 ± 9.4 vs 8.8 ± 4.3 days, *p* = 0.001). There was no difference between CABG and valve-replacement surgery regarding postoperative development of pneumonia (*p* = 0.435).

Pre-operative heart rate was related to postoperative incidence of pneumonia (*p* = 0.047). The percentage of patients with previous COPD and diabetes was greater in the group with postoperative pneumonia. The remaining patients’ characteristics regarding pre-operative variables were similar between the groups.

Whereas none of the intra-operative variables had any effect on development of pneumonia, many postoperative variables were significant risk factors. In patients with postoperative pneumonia, intubation time was longer, postoperative urea, creatinine and potassium levels were higher, more chest tube drainage was encountered, and the need for transfusion of pRBC and FFP was increased.

All variables showing an association (*p* ≤ 0.05) with occurrence of postoperative pneumonia were then entered into a forward stepwise multivariate logistic regression model. The following variables were included in the multivariate model: pre-operative heart rate, previous diabetes, previous COPD, postoperative urea, creatinine and potassium levels, extubation time, number of transfused FFP units, number of transfused pRBC units and postoperative AF. Upon logistic regression analysis of these risk factors, pRBC transfusion, previous COPD and postoperative AF remained as independent predictors for the development of pneumonia post cardiac surgery (Table 2).

**Table 2:** The Outcomes Of Forward Stepwise Binary Logistic Regression And Odds Ratio

*Variables*	*β*	*SE*	*Wald*	*OR (95% CI)*	p-*value*
pRBC transfusion	0.910	0.220	17.131	2.484 (1.614–3.821)	< 0.001
Previous COPD	3.026	0.932	10.530	20.613 (3.315–128.191)	0.001
Postoperative AF	1.732	0.855	4.100	5.653 (1.057–30.228)	0.043

pRBC: packed red blood cells, COPD: chronic obstructive pulmonary disease, AF: atrial fibrillation.

## Discussion

Although cardiac surgery-related mortality has substantially reduced due to advances in surgical techniques and peri-operative care, the incidence of pneumonia post cardiac surgery is still high, varying between 1.5 and 21% in most series.^3^-^8^ Hortal *et al*.^9^ reported a 45.9% incidence of pneumonia in the sub-group of patients needing mechanical ventilation for longer than 48 hours. This wide range in the incidence rates was attributed to the difference in the characteristics of the study population and the diagnostic criteria used to define nosocomial pneumonia.^3^

Surgical technique plays an important role in the occurrence of nosocomial infections. For instance, inadequate haemostasis can lead to hypovolaemia, resulting in an increased need for blood transfusion, inotropic support, duration of surgery, or even possible re-operation. However, besides the surgical technique, several risk factors for pneumonia post cardiac surgery have been identified: age,^4^,^9^ unnecessary use of broad-spectrum antibiotics,^5^,^10^,^11^ duration of mechanical ventilation,^4^-^6^,^9^,^12^,^13^ CPB time,^3^,^9^ re-intubation,^3^,^4^,^9^ emergency surgery,^4^,^5^,^9^ intra-operative inotropic support,^9^ and pre-operative renal dysfunction.^14^

In univariate analysis of our study, duration of mechanical ventilation had a significant effect on postoperative pneumonia, whereas age, gender, CPB time and need for inotropic support had no association with pneumonia. Postoperative but not pre-operative high creatinine and urea levels, indicating renal dysfunction, were more common among patients with pneumonia compared to those without pneumonia. However, multivariate analysis depicted only prior COPD, transfusion of pRBC and postoperative atrial fibrillation as independent risk factors for pneumonia.

COPD was reported to cause postoperative pneumonia in another series.^5^ Nosocomial pneumonia is a frequent event in the course of acute exacerbation of COPD. There is clear evidence that in up to 50% of stable COPD patients, the lower airways are colonised by potential pulmonary pathogens. Advanced age and severity of lung disease are strongly associated with increased risk for pneumonia.

Cardiac surgery, especially CPB, aggravates COPD. Moreover the use of inhaled corticosteroids among patients with COPD significantly increases the risk of developing pneumonia.^15^,^16^ According to Lomas,^17^ inhaled corticosteroid use for at least 24 weeks is associated with a 60 to 70% higher relative risk of pneumonia. Corticosteroid use before elective cardiac surgery may be limited or at least the dose may be decreased in order to decrease the incidence of pneumonia. In addition, immunisation against influenza in older patients with COPD is associated with a 52% reduction in hospitalisations for pneumonia.^18^

The second independent risk factor for the occurrence of pneumonia following cardiac surgery was the need for blood transfusion, consistent with previous reports.^3^-^5^,^8^,^9^ Blood transfusions may cause transient immune suppression, thus increasing the susceptibility to infection. It was found to be an independent risk factor of deep sternal wound infections.^19^

The mechanism of the immunomodulatory effect of allogenic blood transfusion remains elusive. The infusion of foreign antigens in either soluble or cell-associated form has been shown to induce immune suppression, anergy and clonal deletion, most likely mediated by allogenic white blood cells.^8^

Another concern is the altered function of macrophages. After transfusion macrophages lose migratory ability in response to chemotactic stimuli and produce more prostaglandin E2, resulting in decreased activity of antigen-presenting cells and the production of interleukin 2.[Bibr R03]

There is an association between the length of storage of transfused red blood cells and the development of postoperative pneumonia,^3^,^8^ which is seen more rarely among patients with fresh red blood cell transfusions. Various immunosuppressive substances are released from white blood cell granules into red blood cell components during blood storage, contributing to transfusion-induced immunomodulation.^8^ Furthermore, the deleterious effect of stored blood may be due to depleted levels of 2,3-diphosphoglycerate and decreased deformability of stored red blood cells, both impairing oxygen delivery to the tissues.^3^

Recently, the involvement of inflammation in atrial fibrillation has been documented, and high levels of pro-inflammatory proteins, such as C-reactive protein, have been suggested to promote the persistence of atrial fibrillation by inducing structural and/or electrical remodelling of the atria. Atrial biopsies taken from patients in AF have also demonstrated evidence of inflammatory infiltrates within the atrial tissue, with evidence of oxidative damage or occult myocarditis, even among persons who were thought to have had lone AF.^20^

The fact that inflammation plays an important role in the development of both AF and pneumonia may explain the concomitance of these complications after cardiac surgery. It is classic knowledge that pneumonia is one of the non-cardiac causes of AF, predominantly in elderly patients. However, in our study, AF was an independent risk factor for pneumonia post cardiac surgery.

In patients with AF, contraction of the ventricles averages 150/minute. At that rate, the ventricles may not have enough time to fill maximally with blood before the next contraction, particularly without the normal contraction of the atria.^21^ Moreover, the contractility of the ventricle decreases after CPB. Therefore AF decreases the amount of blood pumped by the ventricles and the body begins to compensate by retaining fluid, resulting in the accumulation of fluid in the lungs. Alveolar oedematous fluid is a good culture medium for the development of secondary pneumonia. CPB contributes to this process by aggravating the pulmonary oedema and inflammation.^22^

Also, AF may play an active role in the development of postoperative pneumonia by prolonging the postoperative intubation time. However, postoperative AF has not been suggested as a cause of pneumonia post cardiac surgery in any previous reports. Our findings must be confirmed in larger series and it must be clarified whether pneumonia is only a cause or also a consequence of AF.

The main limitations in our study were the retrospective nature of the analysis and the small sample size, affecting particularly the subgroup with postoperative pneumonia. Therefore any claim about an associative relationship between pre- or peri-operative variables and outcomes should be viewed with caution. The study group was also not treated entirely uniformly, as off-pump CABG patients were included. However, peri-operative care was standardised, which serves to strengthen conclusions on the results.

## Conclusion

On logistic regression analysis, pRBC transfusion, previous chronic obstructive pulmonary disease and postoperative atrial fibrillation remained as independent predictors for the development of postoperative pneumonia. AF particularly should be investigated in future series as a cause of pneumonia.
